# Supplementary material for: Probiotic-Treated Super-Charged NK Cells Efficiently Clear Poorly Differentiated Pancreatic Tumors in Hu-BLT Mice
Source: Cancers (Basel). 2019 Dec 24;12(1):63. doi: 10.3390/cancers12010063 (PMC7017229; doi:10.3390/cancers12010063)
Supplement: Supplementary file 1 [file cancers-12-00063-s001.pdf]

cancers

Article

Probiotic-Treated Super-Charged NK Cells Efficiently Clear Poorly Differentiated Pancreatic Tumors in Hu-BLT Mice

Kawaljit Kaur [1], Anna Karolina Kozlowska [1,2], Paytsar Topchyan [1], Meng-Wei Ko [1], Nick Ohanian [1], Jessica Chiang [1], Jessica Cook [1], Phyu Ou Maung [1], So-Hyun Park [1], Nicholas Cacalano [3,4], Changge Fang [5] and Anahid Jewett [1,3,*]

Results

A. Correlation between NK cell cytotoxicity and the stage of differentiation of MP2 and PL-12 pancreatic tumors and the role of rhTNF-α and rhIFN-γ in induction of differentiation and resistance of MP2 cells to NK cell mediated cytotoxicity

As demonstrated in the main manuscript the stage of differentiation of the tumors was correlated with sensitivity to NK cell mediated cytotoxicity in pancreatic tumors. The highest susceptibility to NK cell mediated cytotoxicity was seen with undifferentiated MP2 tumors whereas the well differentiated PL-12 tumors demonstrated the lowest sensitivity to NK mediated lysis (Supplementary Figure S1A). Stem-like/undifferentiated MP2 and well differentiated Capan pancreatic tumor cells were treated with rhTNF-α and rhIFN-γ and their susceptibility to NK cell mediated lysis was assessed in a standard 4-hour ^{51}Cr release assay. As shown in figure S1B, the combination of rhTNF-α and rhIFN-γ were able to upregulate CD54, MHC-1 and B7H1 and down modulate CD44 in MP-2 tumors. Both rhTNF-α and rhIFN-γ were able to increase surface expression of CD54 and MHC-class I, however, only rhIFN-γ was able to upregulate B7H1 (Supplementary Figure S1B). The addition of rhTNF-α to MP2 was able to induce moderate resistance against NK cell mediated cytotoxicity whereas rhIFN-γ induced significant resistance (Supplementary Figure S1C). As expected there was less lysis of Capan tumors by the NK cells and treatment with rhIFN-γ and rhTNF-α induced moderate resistance in these cells (Supplementary Figure S1C).

B. Reconstitution of human immune system in hu-BLT mice and decreased frequencies of NK cells in hu-BLT mice as compared to humans

Hu-BLT mice that were reconstituted with the human immune system, exhibited greater than 90% reconstitution with huCD45+ immune cells in different tissue compartments (Supplementary Figsure S2B,C). Similar to humans in which a range of frequencies can be seen in peripheral blood NK cells between donors, there are also variable percentages of NK cells in peripheral blood of hu-BLT mice reconstituted with different donor immune cells. Based on the number of hu-BLT mice tested so far, on average there is a lower percentage of NK cells in peripheral blood of hu-BLT mice as compared to human donor peripheral blood (Supplementary Figure S2D). Similar percentages of T cell subsets between human and hu-BLT mice in peripheral blood were found (Supplementary Figure S2D).

C. Frequencies of Immune Subsets in the Pancreas

The majority of infiltrating human immune cells in the pancreas were CD3+T (54%) and B cells (43.3%), with CD8+T cells constituting the larger proportions of the T cells (approximately 80%) than CD4+ T cells (approximately 20%) (Supplementary Figure S2E). NK and CD14+ cells constituted minor subpopulations of immune cells in the pancreas of healthy hu-BLT mice (Supplementary Figure S2E).

A

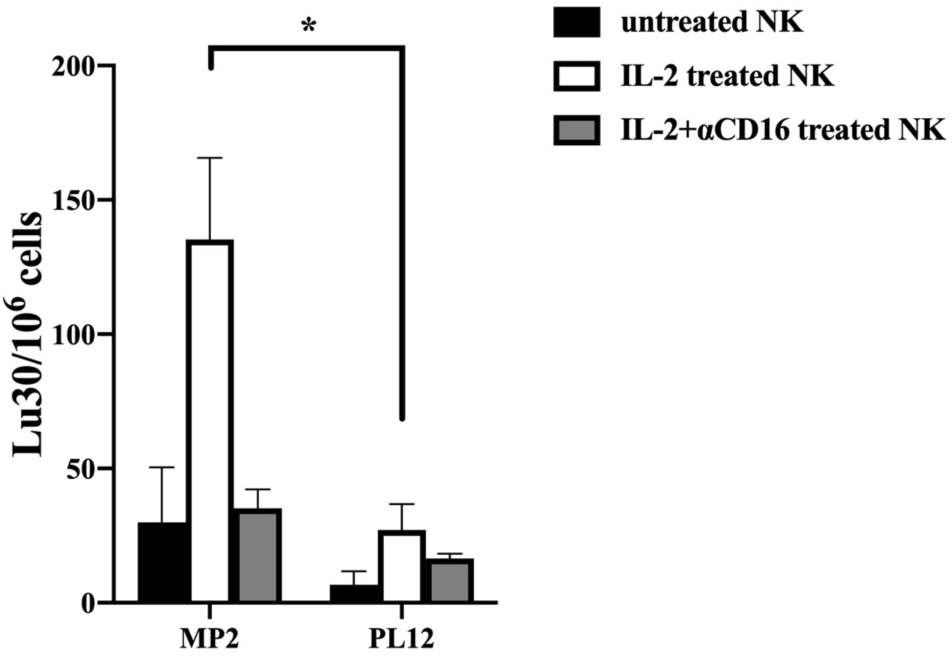
*
untreated NK
IL-2 treated NK
IL-2+αCD16 treated NK
Lu30/10⁶ cells
200
150
100
50
0
MP2
PL12

B

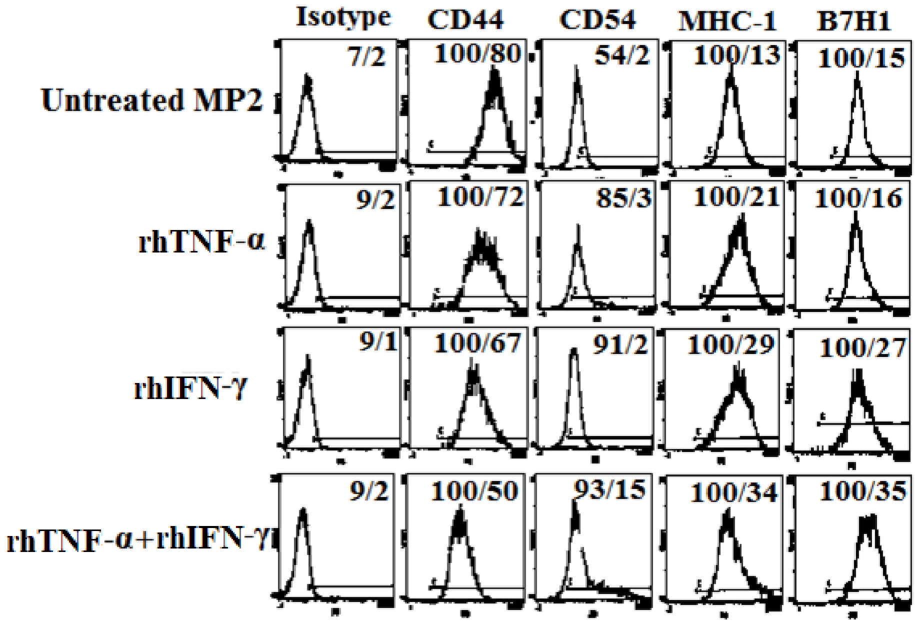
Isotype
CD44
CD54
MHC-1
B7H1
Untreated MP2
rhTNF-α
rhIFN-γ
rhTNF-α+rhIFN-γ
7/2
100/80
54/2
100/13
100/15
9/2
100/72
85/3
100/21
100/16
9/1
100/67
91/2
100/29
100/27
9/2
100/50
93/15
100/34
100/35

C

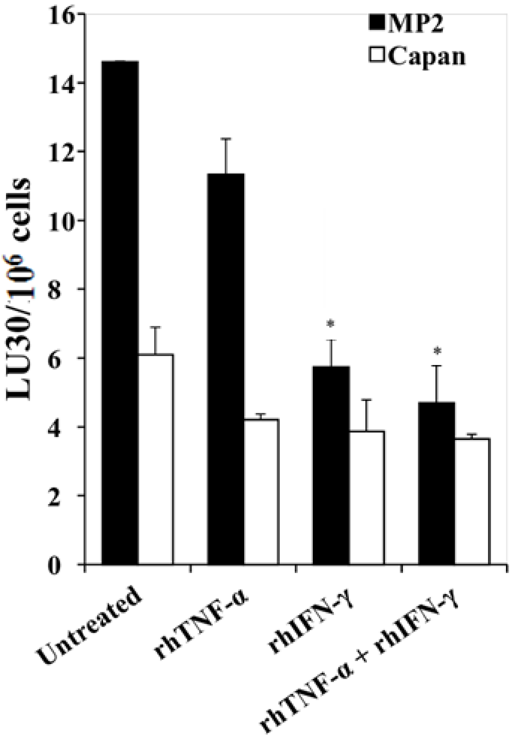

Figure S1. The stage of differentiation in pancreatic tumors correlated with susceptibility to NK cell-mediated cytotoxicity and combination of rhTNF-*α* and rhIFN-*γ* induce differentiation and resistance of MP2 cells to NK cell-mediated cytotoxicity. Freshly isolated NK cells were left untreated or treated with IL-2 (1000 U/mL) or the combination of anti-CD16 mAb (3 µg/mL) and IL-2 (1000 U/mL) for 18 h before they were used in co-cultures with ^{51}Cr labeled MP2, and PL12. NK cell-mediated cytotoxicity was determined using 4-hour ^{51}Cr release assay, and the lytic units 30/10^6 cells were determined using inverse number of NK cells required to lyse 30% of the target cells ×100 (n = 5 independent experiments). (**A**). Cells were left untreated or treated with rhTNF-*α* (20 ng/mL), rhIFN-*γ* (200 U/mL) or the combination of rhTNF-*α* (20 ng/mL) and rhIFN-*γ* (200 U/mL) for 24 h. Afterwards, the cells were detached and the surface expression of CD44, CD54, MHC-class I and B7H1 were assessed using staining with PE conjugated antibodies followed by flow cytometric analysis. Isotype control antibodies were used as controls (**B**) MP2 and Capan cells were treated as described in Fig. S1A, and were detached from the tissue culture plates, labeled with ^{51}Cr and used in a standard 4-hour ^{51}Cr release assay using IL-2 (1000 U/mL) treated NK cells. Pre-treatment of NK cells with IL-2 (1000 U/mL) were carried out for 18-24 h. Percent cytotoxicity was determined at different effector to target ratio and the lytic units 30/10^6 cells were determined using inverse number of NK cells required to lyse 30% of the tumor cells ×100 (**C**) One of the eight representative experiments is shown in the figure.

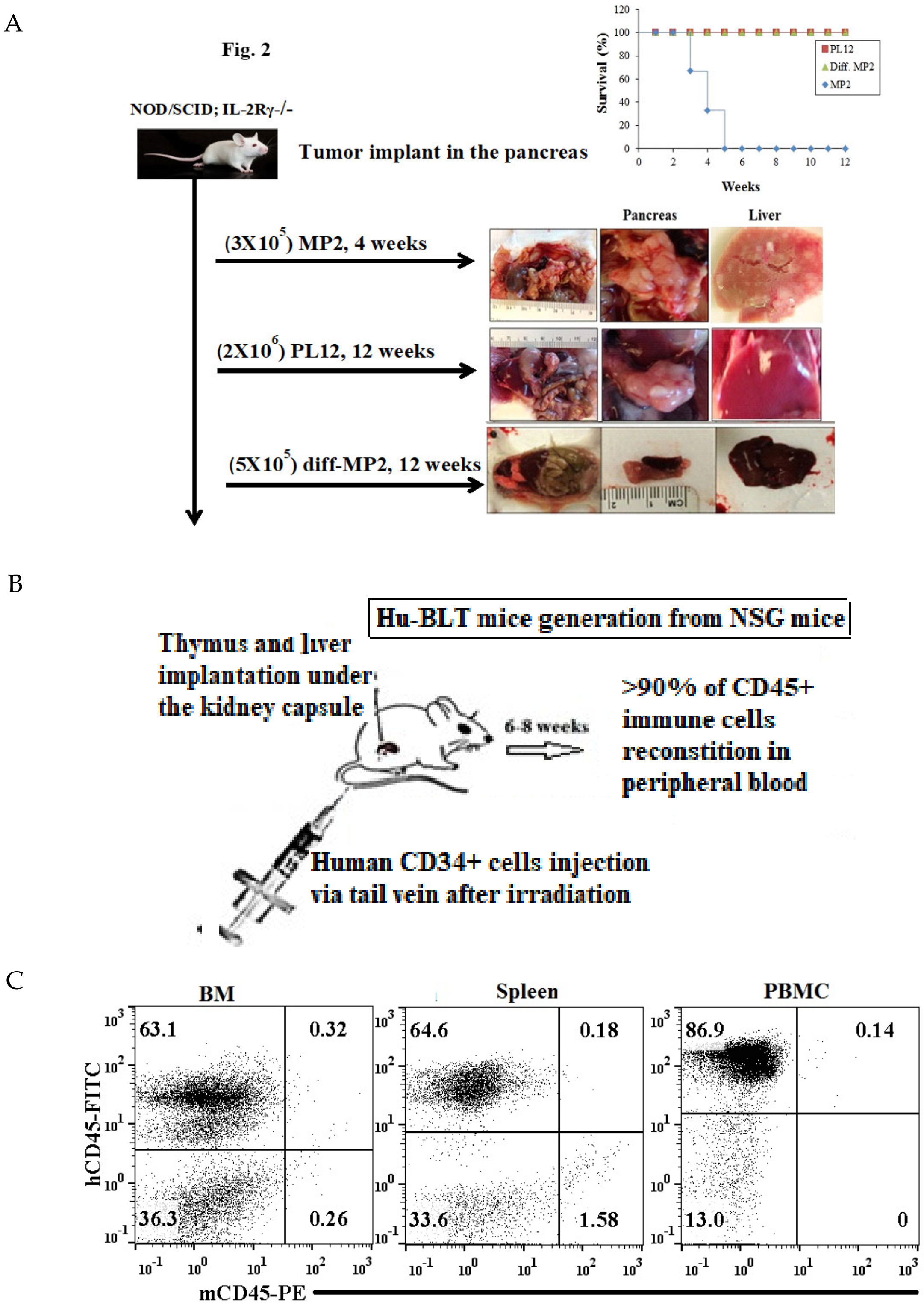
A
Fig. 2
NOD/SCID; IL-2Rγ-/-
Tumor implant in the pancreas
Survival (%)
Weeks
PL12
Diff. MP2
MP2
Pancreas
Liver
(3X10^5) MP2, 4 weeks
(2X10^6) PL12, 12 weeks
(5X10^5) diff-MP2, 12 weeks
B
Hu-BLT mice generation from NSG mice
Thymus and liver implantation under the kidney capsule
6-8 weeks
>90% of CD45+ immune cells reconstition in peripheral blood
Human CD34+ cells injection via tail vein after irradiation
C
BM
Spleen
PBMC
63.1
0.32
36.3
0.26
64.6
0.18
33.6
1.58
86.9
0.14
13.0
0
hCD45-FITC
mCD45-PE

D

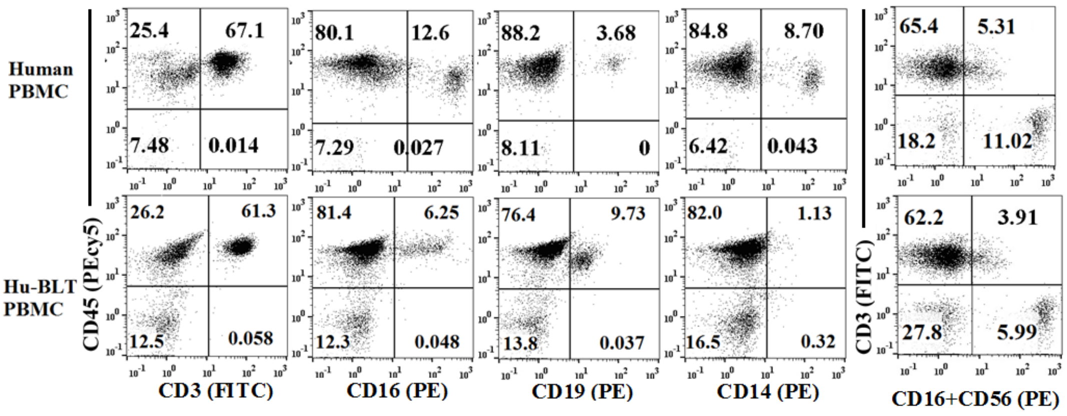
Human PBMC
Hu-BLT PBMC
CD45 (PEcy5)
CD3 (FITC)
25.4 67.1 7.48 0.014
80.1 12.6 7.29 0.027
88.2 3.68 8.11 0
84.8 8.70 6.42 0.043
65.4 5.31 18.2 11.02
26.2 61.3 12.5 0.058
81.4 6.25 12.3 0.048
76.4 9.73 13.8 0.037
82.0 1.13 16.5 0.32
62.2 3.91 27.8 5.99
CD3 (FITC)
CD16 (PE)
CD19 (PE)
CD14 (PE)
CD16+CD56 (PE)

E

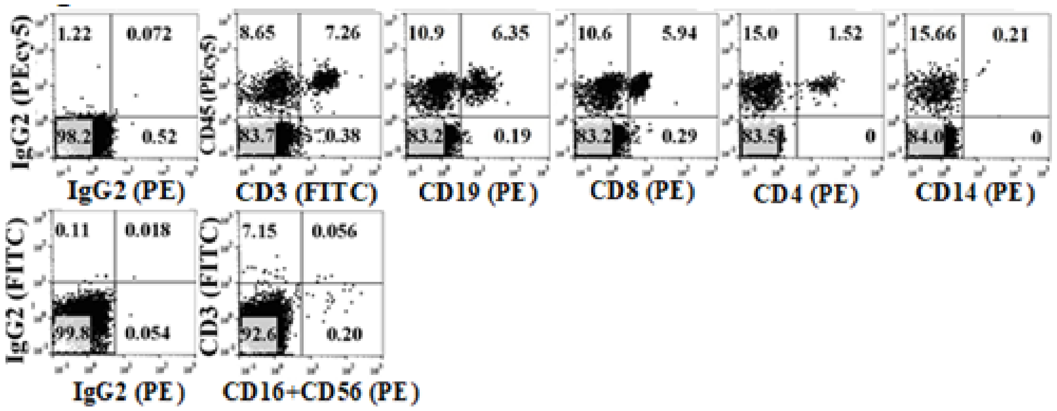
IgG2 (PEcy5)
CD45 (PEcy5)
1.22 0.072 98.2 0.52
8.65 7.26 83.7 0.38
10.9 6.35 83.2 0.19
10.6 5.94 83.2 0.29
15.0 1.52 0
15.66 0.21 84.0 0
IgG2 (PE)
CD3 (FITC)
CD19 (PE)
CD8 (PE)
CD4 (PE)
CD14 (PE)
IgG2 (FITC)
CD3 (FITC)
0.11 0.018 99.8 0.054
7.15 0.056 92.6 0.20
IgG2 (PE)
CD16+CD56 (PE)

F

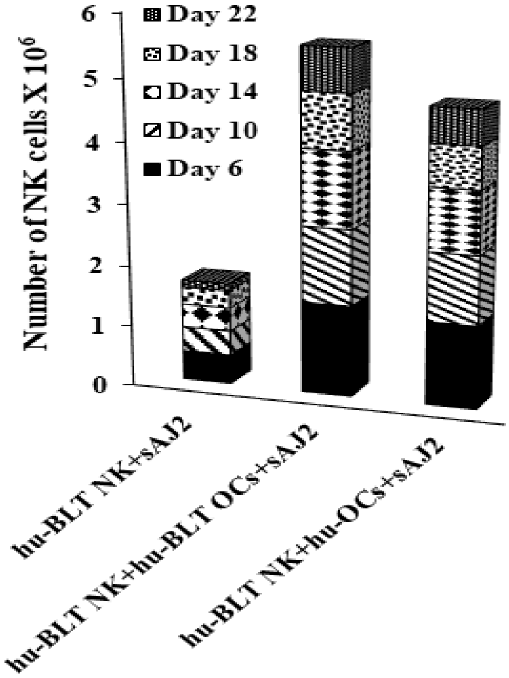
Number of NK cells X 10^6
0
1
2
3
4
5
6
Day 22
Day 18
Day 14
Day 10
Day 6
hu-BLT NK+sAJ2
hu-BLT NK+hu-BLT OCs+sAJ2
hu-BLT NK+hu-OCs+sAJ2

G

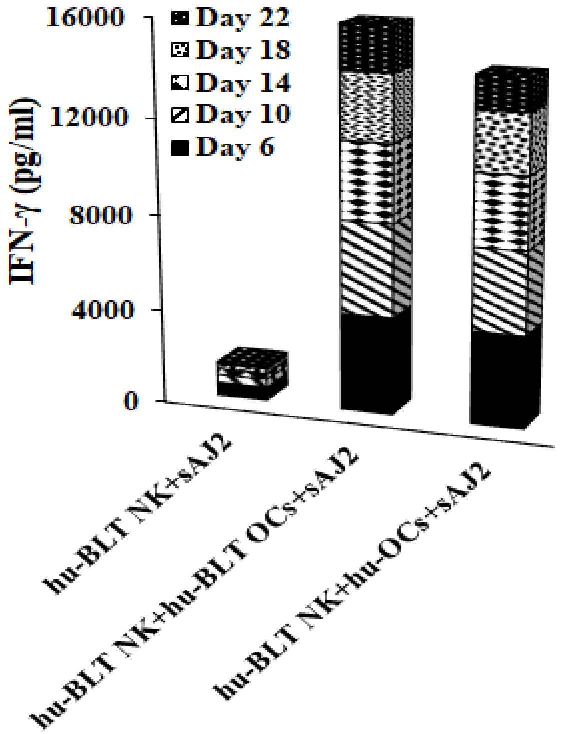

Figure S2. Phenotypic characteristics of bone marrow, spleen, peripheral blood, pancreas in hu-BLT mice. Lack of tumor growth, metastasis and long-term survival of NSG mice after orthotopic implantation of NK-supernatant differentiated MP2 tumors in pancreas. MP2 tumors were differentiated by the NK-supernatants as described in the Materials and Method section. Patient-derived differentiated PL12 (2×10^6) ($n = 3$), NK-differentiated MP2 tumors (diff-MP2) (5×10^5) ($n = 3$), and MP2 tumors (3×10^5) ($n = 3$) were implanted into the pancreas of NSG mice. The rates of survival of the mice were assessed (top right corner), and tumor growth in the pancreas as well as tumor metastasis to liver were determined after euthanasia (**A**). Hu-BLT mice were generated as depicted in Figure (**B**) and described in Materials and Methods section. Reconstitution of human immune system was analyzed in PBMCs, bone marrow and splenocytes using flow cytometric analysis after staining with anti-human CD45 and anti-mouse CD45 antibodies (one of six representative experiment is shown in the figure). The percentages of human and mouse CD45+ immune cells were determined by staining with respective antibodies followed by flow cytometric analysis (**C**). PBMCs were isolated from hu-BLT mice and human donors as described in Materials and Methods section and percentages of CD3, CD16, CD56, CD19, and CD14 within human CD45+ immune-cells were determined using antibody staining followed by flow cytometric analysis (one of six representative experiment is shown in the figure) (**D**). Hu-BLT pancreas were harvested, single cells suspension was obtained as described in Materials and Methods section and percentages of CD3, CD19, CD8, CD4, CD16, CD56 and CD14 within human CD45+ immune-cells in pancreas were determined using antibody staining followed by flow cytometric analysis (one of six representative experiment is shown in the figure) (**E**). OCs were generated from hu-BLT bone marrow monocytes and human peripheral blood monocytes as described in Material and Methods section. NK cells purified from hu-BLT splenocytes were pre-treated with IL-2 (1000 U/mL) and anti-CD16mAb (3 μg/mL) for 18 hours and then either cultured alone or with hu-BLT-OCs or human OCs in the presence of sAJ2 (NK: OCs: sAJ2; 2:1:4) and the numbers of expanding NK cells were counted on days 6, 10, 14, 18 and 22. At each day of culture equal numbers of NK cells from each group were cultured and cell growth determined (**F**). The supernatants from the NK cells and OCs cultures in the presence of sAJ2, as described in Fig. S2E were harvested on days 6, 10, 14, 18 and 22, the levels of IFN-γ were determined using single ELISA (**G**).

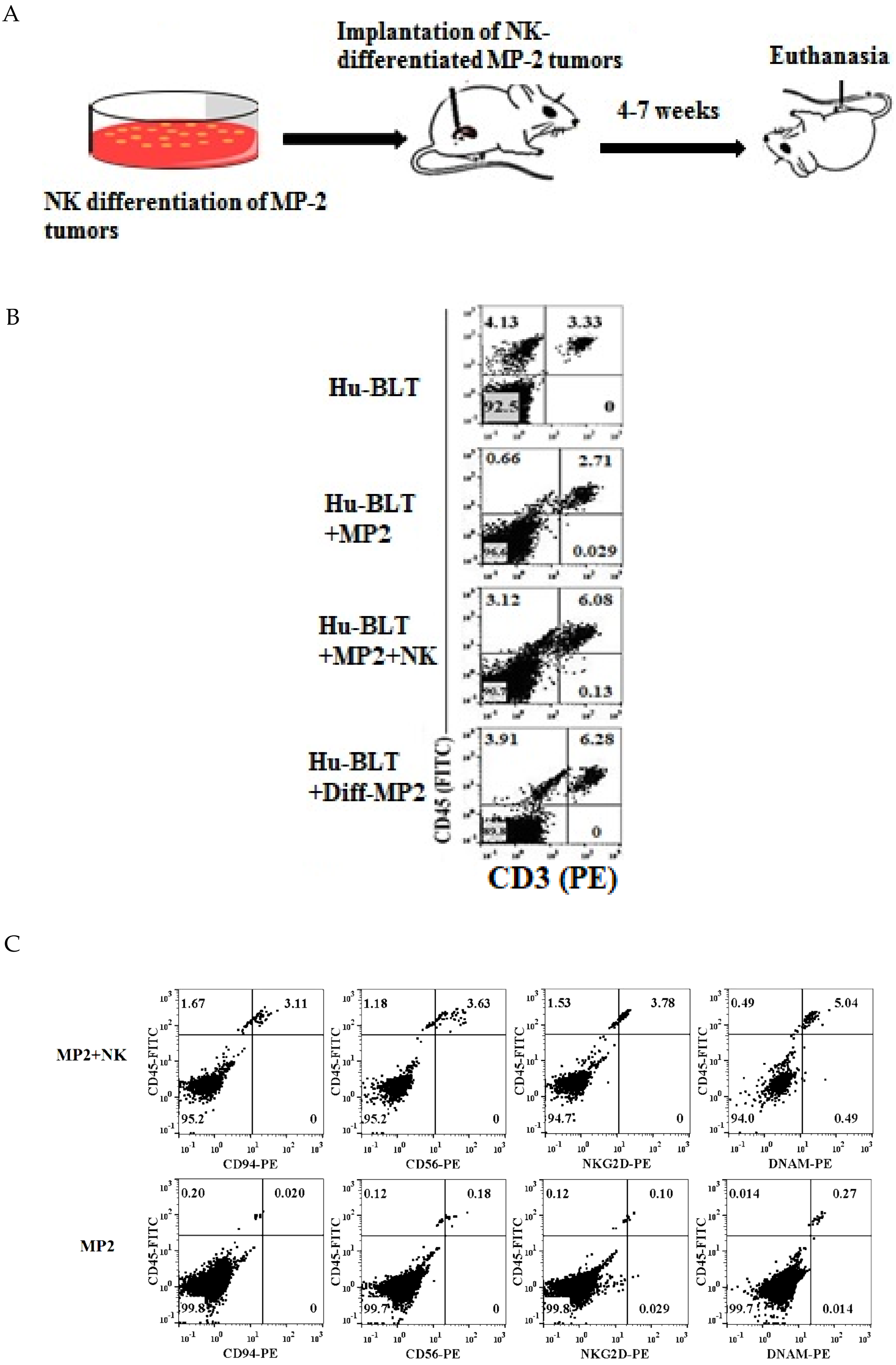
A
Implantation of NK-differentiated MP-2 tumors
Euthanasia
4-7 weeks
NK differentiation of MP-2 tumors
B
Hu-BLT
Hu-BLT +MP2
Hu-BLT +MP2+NK
Hu-BLT +Diff-MP2
CD45 (FITC)
CD3 (PE)
4.13
3.33
92.5
0
0.66
2.71
0.029
3.12
6.08
0.13
3.91
6.28
0
C
MP2+NK
MP2
CD45-FITC
CD94-PE
CD56-PE
NKG2D-PE
DNAM-PE
1.67
3.11
95.2
0
1.18
3.63
95.2
0
1.53
3.78
94.7
0
0.49
5.04
94.0
0.49
0.20
0.020
99.8
0
0.12
0.18
99.7
0
0.12
0.10
99.8
0.029
0.014
0.27
99.7
0.014

D

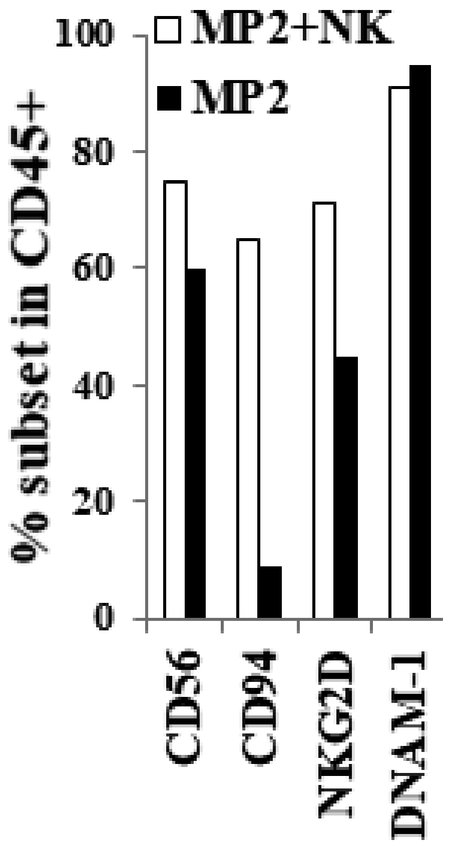

Figure S3. Single injection of super-charged NK cells inhibited tumor growth and increased immune cells in the pancreas in hu-BLT mice. Hu-BLT mice were generated as described in Materials and Methods section. MP2 tumors were differentiated by the NK-supernatants as depicted in the figure (**A**). Hu-BLT mice were implanted with MP-2 (1 × 10⁶ cells) tumors in the presence and absence of super-charged NK cell injection and NK-differentiated-MP2 tumor cells (1 × 10⁶ cells) in the pancreas and disease progression was monitored for another 4–7 weeks (*n* = 3 per each experimental condition). The pancreas were harvested postmortem, and percentages of CD3+ cells within human CD45+ immune cells from the pancreas were determined using antibody staining followed by flow cytometric analysis (*n* = 3 per each experimental condition) (**B**). Hu-BLT mice were implanted with MP-2 (1 × 10⁶ cells) tumors in the pancreas in the presence and absence of super-charged NK cell injection (IV) and disease progression was monitored for another 4–7 weeks. Tumors were resected, and single cell cultures were prepared and cultured for 7 days, after which percentages of human CD45, CD94, CD56, NKG2D, and DNAM within the tumors were determined after staining with antibodies, followed by flow cytometric analysis (C). The percentages of each of CD56, CD94, NKG2D and DNAM was calculated within CD45+ cells D).

A

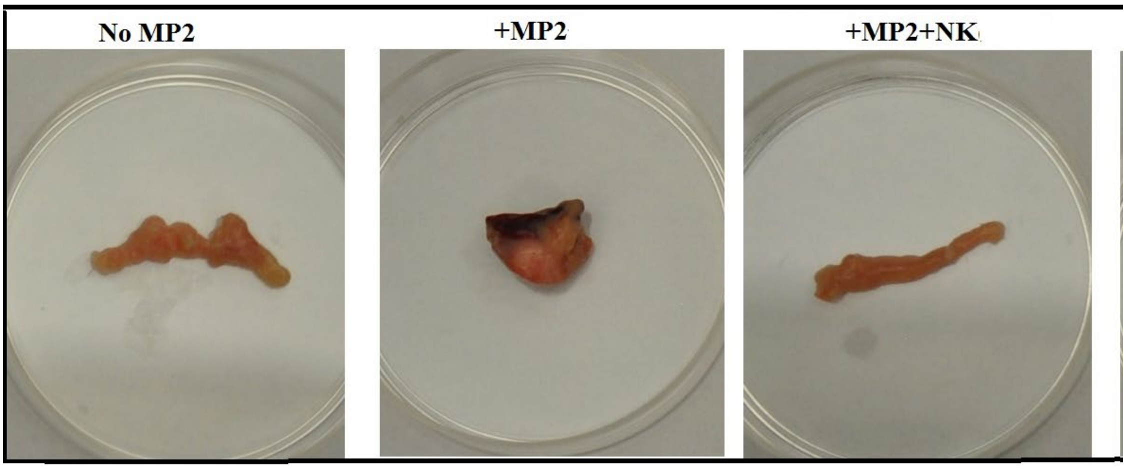

B

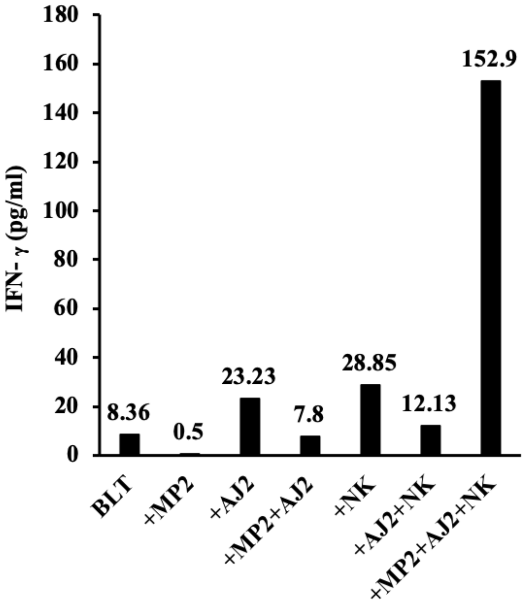

C

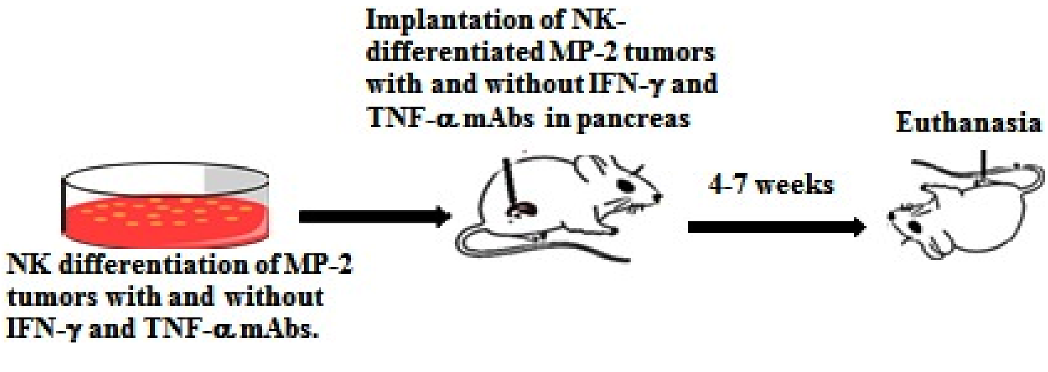

Figure S4. Single injection of super-charged NK-cells with/without feeding with AJ2 inhibited tumor growth due to differentiation of tumors in hu-BLT mice. Hu-BLT mice were implanted with 1 × 10^6 tumor cells in the pancreas, and after 1–2 weeks mice received 1.5 × 10^6 super-charged NK cells via tail vein injection, and disease progression was monitored for another 3–5 weeks. Mice were also fed AJ2 (5 billion/dose) starting 1–2 weeks before tumor implantation, and thereafter every 48 h throughout the experiment. At the end of experiment, mice were sacrificed, and pancreas/pancreatic tumor pictures were taken postmortem (n = 9 per each experimental condition) (**A**). Implantation of tumor cells in the pancreas and tail vein injection of super-charged NK cells were carried out as

depicted in Supplementary Figure S4A, and at the time of sacrifice mice were bled and the levels of IFN-γ in the serum were determined using multiplex array (*n* = 3) (**B**). Highly purified healthy human NK cells were treated with IL-2 (1000 U/mL) and anti-CD16 mAb (3 μg/mL) for 18 h, after which the supernatants were collected and added to MP2 tumors in the presence/absence of anti-TNF-α (1:100) and anti-IFN-γ (1:100) for a period of 5 days. Hu-BLT mice were implanted with diff-MP2 (1 × 10^6 cells) or diff-MP2 treated with monoclonal antibodies against INF-γ and TNF-α (1 × 10^6 cells) and disease progression was monitored for another 4–7 weeks (*n* = 6 per each experimental condition) (**C**).

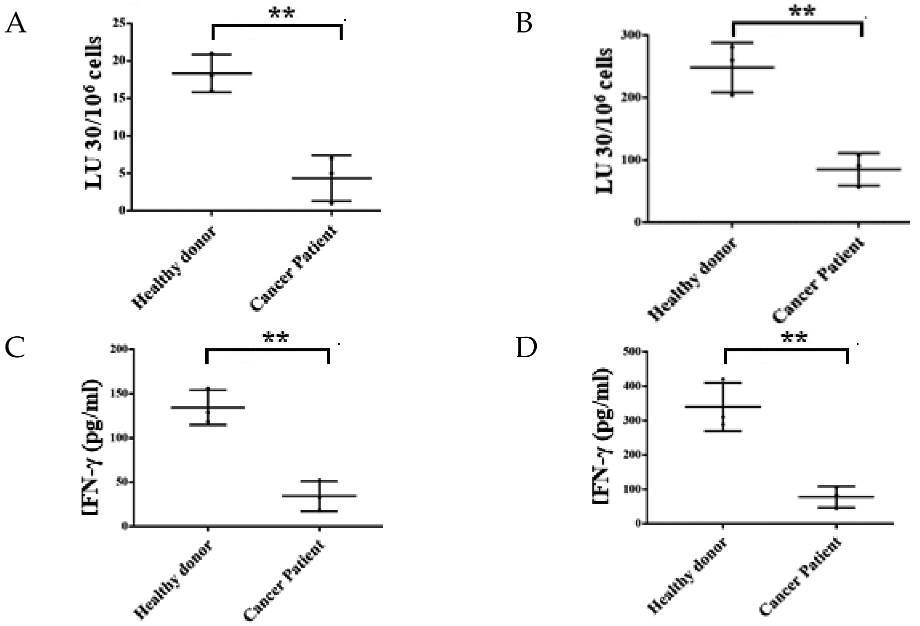

Figure S5. NK cell cytotoxicity and ability to secrete IFN-γ is severly decreased in pancreatic cancer patients. PBMCs (**A**) and purified NK cells (**B**) from healthy human donors and pancreatic cancer patients were obtained and treated with IL-2 (1000 U/mL) for 18 h before they were used for cytotoxicity against OSCSCs using 4 h ^{51}Cr release assay. The lytic units (LUs) 30/10^6cells were determined using inverse number of NK cells required to lyse 30% of the tumor-cells ×100 (*n* = 3 for each experimental condition) (**A** and **B**) PBMCs (**C**) and purified NK cells (**D**) from healthy human donors and pancreatic cancer patients were treated with IL-2 (1000 U/mL) for 18 h before the supernatants were harvested and IFN-γ secretion was determined using ELISA (*n* = 3 for each experimental condition) (**C** and **D**).

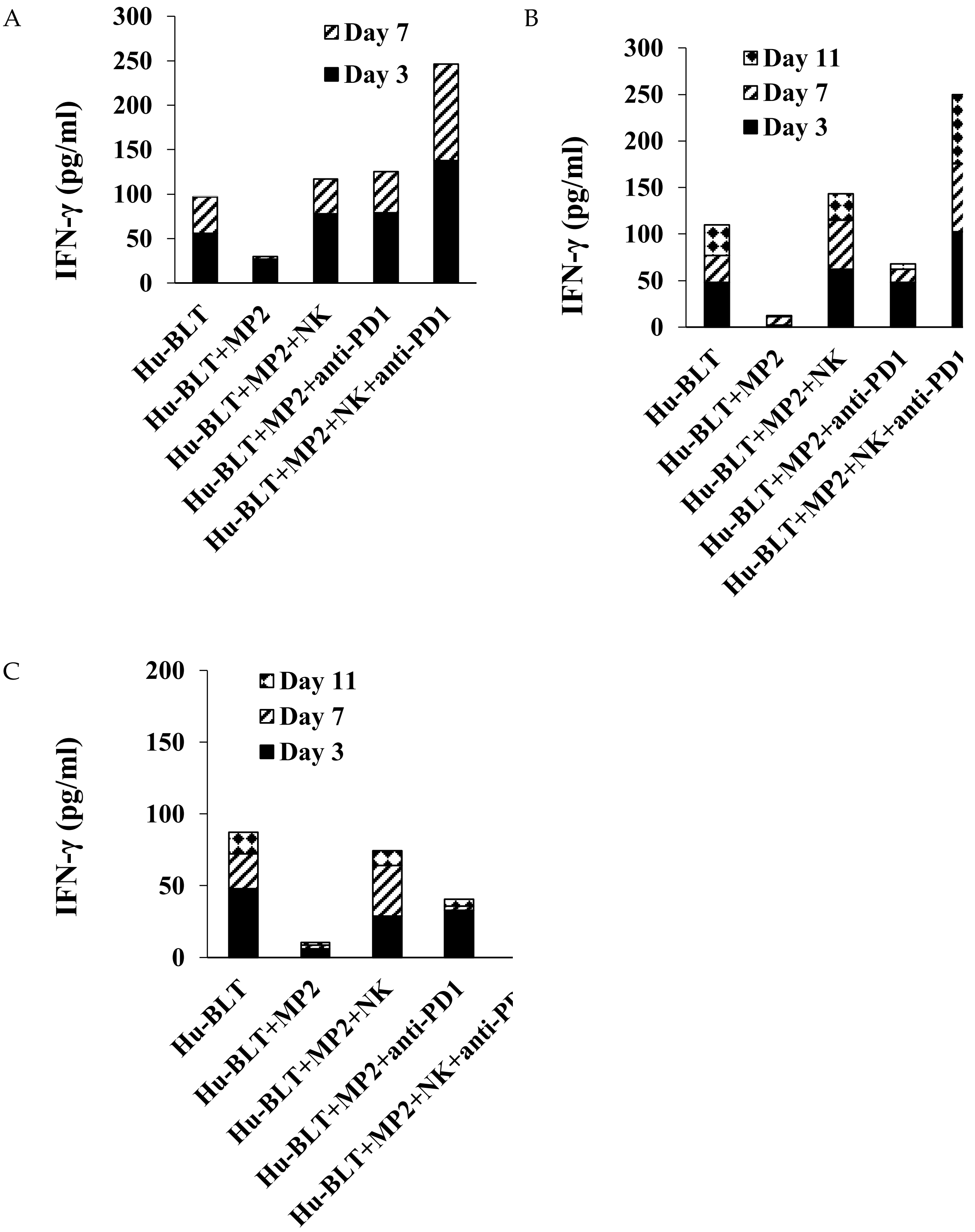

Figure S6. Combination of super-charged NK cells with anti-PD1 antibody injection increased IFN-γ secretion substantially by PBMCs, splenocytes and bone marrow derived immune cells and halted growth of poorly differentiated MP2 tumors in hu-BLT mice. Successfully reconstituted hu-BLT mice were orthotopically injected with 1×10^6 of human MP2 cells in the pancreas. One or two weeks after tumor implantation hu-BLT mice received 1.5×10^6 super-charged NK cells via tail vein injection. Seven days later, anti-PD1 (50 μg/mice) was injected via tail vain injection. At the end of experiment, animals were sacrificed and bone marrows were harvested from hu-BLT mice and single cell suspensions were obtained as described in Materials and Methods section. Cells were treated with IL-2 (1000 U/mL), on days as specified in the figures and supernatants were harvested from cultures and IFN-γ secretion was determined using single ELISA (**A**). NK enriched cells (**B**) and CD3+ T cells (**C**)

isolated from hu-BLT splenocytes were treated with IL-2 (1000 U/mL and 100 U/mL respectively), on days as specified in the figures and supernatants were harvested from cultured cells and IFN-γ secretion was determined using single ELISA (**B** and **C**). One of the two representative experiments is shown in the figure.

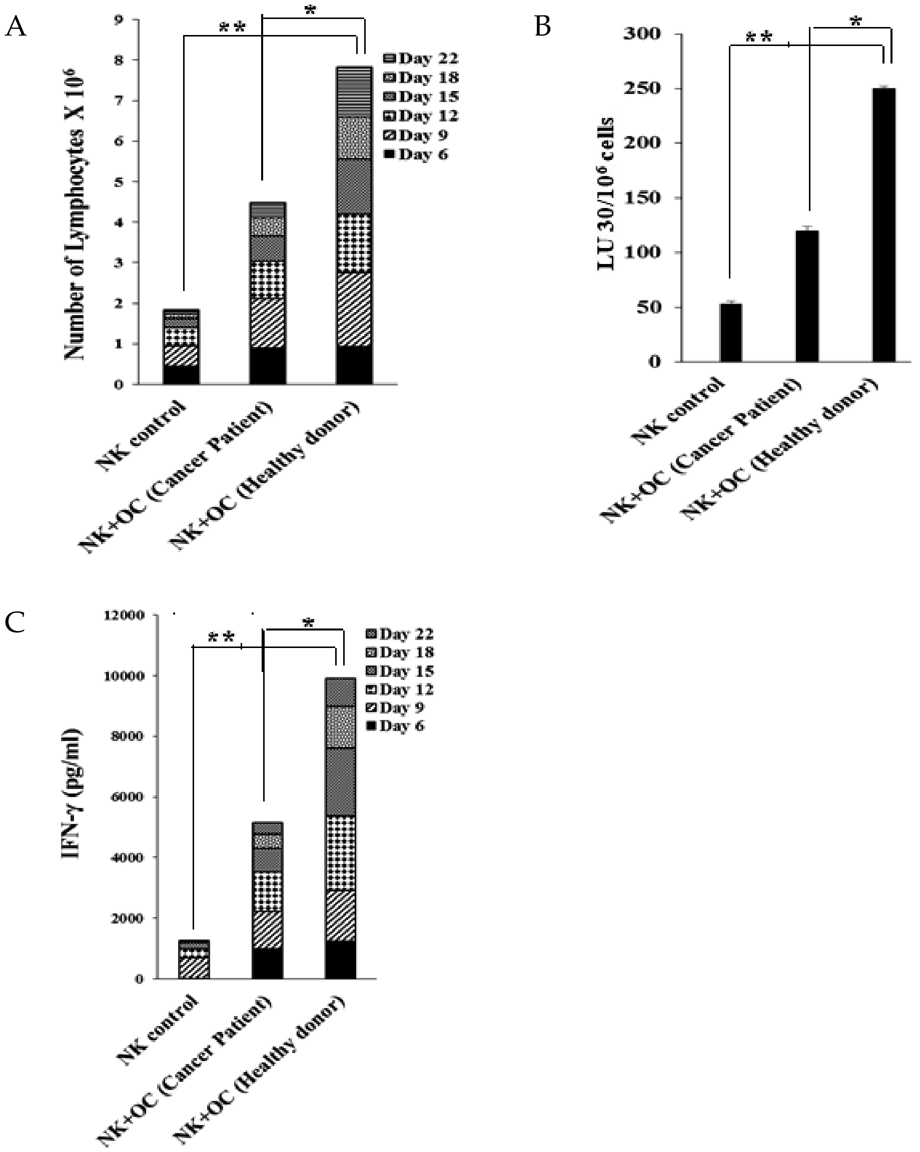

Figure S7. OCs from pancreatic cancer patients expanded lower numbers of super-charged NK cells and generated NK cells secreted lower levels of IFN-γ when compared to healthy individuals. OCs were generated from the peripheral blood-derived monocytes of healthy human-donors and pancreatic cancer patients, as described in the Material and Methods section and were cultured with healthy human NK cells in the presence of sonicated AJ2 (sAJ2) and the numbers of NK cells were counted on days 6, 9, 12, 15, 18 and 22. On each day of culture, equal numbers of NK cells from each group were cultured and cell growth was determined (**A**). OCs were generated from the peripheral blood-derived monocytes of healthy human-donors and pancreatic cancer patients, as described in the Material and Methods section and were cultured with healthy human NK cells in the presence of sAJ2, and the numbers of NK cells were counted on days 6, 9, 12, 15, 18 and 22. On each day of culture, equal numbers of NK cells from each group were cultured. On day 15 of culture, NK cells were counted, and equal numbers of NK cells were used for cytotoxicity against OSCSCs using 4-hour ^{51}Cr release assay. The lytic units (LUs) 30/10^6cells were determined using inverse number of NK cells required to lyse 30% of the tumor-cells ×100 (**B**). Supernatants from the cultures were harvested on

days 6, 9, 12, 15, 18 and 22 and the levels of IFN-γ were determined using ELISA (**C**). One of three representative experiments is shown (**A**–**C**).

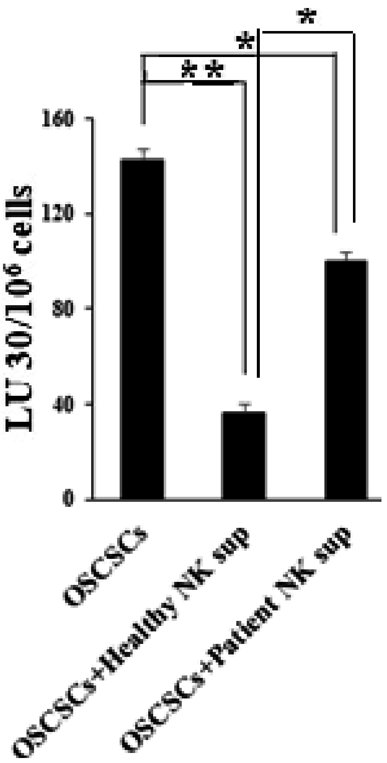

Figure S8. Identical amounts of IFN-γ secreted by cancer patients NK cells in comparison to healthy donor NK cells induce lower levels of differentiation in oral cancer stem-like tumors. Supernatants containing equal amounts of IFN-γ from the healthy donor and pancreatic patients' NK cells treated with IL-2 (1000 U/mL) and anti-CD16 mAb (3 ◎g/mL) for 18 h were added to OSCSCs for 4 days, to induce differentiation. Allogeneic NK cells from healthy human donors were treated with IL-2 (1000 U/mL) for 18–24 h before they were used in cytotoxicity against untreated and healthy and patient NK-supernatant differentiated OSCSCs. Tumors were ^{51}Cr labeled and used in the cytotoxicity assay, and the lytic unit (LUs) 30/10^6 were determined using inverse number of NK cells required to.
